# Gastrointestinal Symptoms and the Assessment of Diet in Shift Workers: A Systematic Scoping Review

**DOI:** 10.1111/jhn.70114

**Published:** 2025-08-28

**Authors:** Seham H. Alyami, Miranda C. E. Lomer, Rachel Gibson

**Affiliations:** ^1^ Department of Nutritional Sciences, School of Life Course & Population Sciences King's College London London UK; ^2^ Department of Nutrition and Dietetics Guy's and St Thomas' NHS Foundation Trust London UK

**Keywords:** diet, dietary behaviours, occupational health, gastrointestinal symptoms, shift work

## Abstract

**Background:**

Shift work is essential in the modern economy. However, it has been associated with adverse health outcomes, including gastrointestinal (GI) symptoms. This systematic scoping review aimed to identify current evidence on GI symptoms and dietary intake and behaviours among shift‐working populations.

**Methods:**

A systematic search was conducted in March 2024 across five databases (MEDLINE Ovid, PubMed, Scopus, CINAHL, clinical trial registers and pre‐print) using the Population, Context, and Concept (PCC) framework. Two reviewers independently screened and extracted data. Study characteristics were summarised using narrative and quantitative synthesis approaches.

**Results:**

Forty‐one articles met the inclusion criteria; 87.8% (*n* = 36) articles were cross‐sectional. Most articles focused on nurses (*n* = 27, 65.9%), with night and rotating shifts being the most examined shift schedules. Articles reported on general GI symptoms (*n* = 8, 19.5%), Irritable Bowel Syndrome (IBS) (*n* = 3, 7.3%), and constipation (*n* = 3, 7.3%), with most showing positive associations between GI symptoms and shift work. A range of self‐reporting tools were used to assess GI symptoms (*n* = 23) and dietary intake (*n* = 9). Only 12 articles (29.3%) assessed dietary behaviours, all of which relied on self‐reported measures, with limited detail on the timing of meals, and fluid or fibre intake.

**Conclusion:**

This review found that research on GI symptoms and the assessment of dietary intake and behaviours in shift workers is limited and inconsistent. Research is needed to better assess GI symptoms, and time‐specific dietary assessment tools.

## Summary

- Evidence links shift work with a higher prevalence of general gastrointestinal symptoms, including irritable bowel syndrome and constipation, although the role of diet is unclear.
- This review found, that despite dietary modification being a frontline approach for managing gastro intestinal symptoms, few articles assessed dietary intake in relation to symptoms.
- Key limitations identified in the review included inconsistent definitions of shift work, limited use of validated assessment tools for symptoms and diet intake, and a lack of representation across occupational groups.

## 1 Introduction

Shift‐based employment is widespread across global labour markets, with its prevalence varying by country and industry. In the United Kingdom, approximately 27% of the workforce is employed in roles involving shift‐based schedules [1]. In the United States, an estimated 25% of workers follow non‐standard working hours, including evening, night, or rotating shifts [2]. In Australia, 16% of the workforce regularly engages in shift work [3]. Shift work encompasses various schedules, including night shifts, rotating shifts, and on‐call duties. Although no standardised definition of shift work or shift exposure currently exists [4], it is generally defined as employment occurring outside the typical ‘9:00 AM to 5:00 PM’ workday [5]. In the United Kingdom, night shift work is defined as working a minimum of 3 h between 11:00 PM and 6:00 AM [6], with around 12% of the UK workforce engaged in night work [7]. Shift work is prevalent across various sectors, such as healthcare and transportation [6], where continuous operations require flexible scheduling patterns, including fixed, rotating, and split shifts. While shift work may offer increased flexibility and earning potential, it also introduces significant challenges, such as disrupted sleep patterns and adverse health outcomes, which require effective management strategies [8].

Night shift work has been associated with long‐term health risks, including increased risks of cardiovascular diseases [9], type 2 diabetes [10], and gastrointestinal (GI) disorders [11]. Although these conditions develop with prolonged exposure, shift workers frequently report acute symptoms, such as GI discomfort, during or following shifts, raising concerns about short‐term health impacts [12]. This highlights the need to better understand the prevalence and types of GI symptoms among shift workers and how diet may contribute to or mitigate these effects.

The GI system is vulnerable to circadian disruptions, as its functions include gastric secretion, bile acid production, bowel movements, and immune activity, all of which are closely tied to circadian rhythms [13, 14]. Misalignment of these rhythms, combined with nocturnal eating habits often observed in shift workers, can impair GI function and metabolic health [15]. As a result, shift workers frequently experience GI symptoms such as abdominal pain, bloating, and indigestion [16]. Compared to individuals with standard working hours, shift workers exhibit more irregular eating patterns [17, 18], increased night‐time snacking [18], and higher consumption of sugar‐sweetened beverages [19, 20], and saturated fats [20]. Shift work also disrupts meal timing, which may impair metabolic processes and overall health [21, 22]. These disruptions are hypothesised to result from circadian misalignment affecting peripheral cellular clock mechanisms in the liver, pancreas, and GI tract due to altered fasting–feeding cycles [22].

Although some evidence links shift work to GI symptoms, there is limited research examining the role of diet in these symptoms. This systematic scoping review aimed to identify and characterise articles conducted among shift workers that investigate GI symptoms and to assess how diet was measured in these articles. The findings will inform the feasibility of future systematic reviews, improve the understanding of the interplay between diet and GI symptoms in shift workers, and identify key research gaps.

## 2 Methods

A scoping review was conducted based on the Joanna Briggs Institute (JBI) methodology [23], and the Preferred Reporting Items for Systematic Reviews and Meta‐analyses extension (PRISMA‐ScR) [24]. A scoping review was deemed appropriate due to the heterogeneity in definitions of shift work and diversity of occupational groups in the existing literature [25]. This approach enabled a broad examination of research to identify key gaps. The protocol was registered on the Open Science Framework (OSF) on 25 March 2024 and is publicly available [26].

### 2.1 Search Strategy and Articles Selection

The Population, Concept, and Context (PCC) framework was used, in line with the JBI methodology, to construct search terms and determine eligibility criteria [23]. The population included adults ( > 18 years old) who work night shifts, rotating shifts, fixed shifts, daytime shifts, or articles conducted in adults undergoing a simulated shift work design. All occupations were considered; however, articles were excluded if shift work was not explicitly specified.

The primary concept was all types of GI symptoms, and any tool used for assessment without limitations. Articles reporting GI diseases, e.g., inflammatory bowel diseases (Chron's or ulcerative colitis), bowel cancers, GI surgery, or articles conducted on animals were excluded. Articles that didn't mention shift work exposure were also excluded. The secondary concept was diet, which includes all dietary behaviours such as nutrient intake, food and beverage choices, amount serving sizes, time, and frequency of eating.

The context was explored through a scoping review, which was not restricted by specific types of evidence. No date restriction was applied to the search to provide an inclusive overview of the literature and identify any gaps in the evidence for future research. The detailed inclusion and exclusion criteria are in the Supporting Information (Table S1). The following research questions guided this review: (i) What types of articles have investigated GI symptoms among shift workers? (ii) What GI symptoms and dietary behaviours have been reported in shift‐working populations, and how frequently are these examined together? and (iii) What methods have been used to assess GI symptoms and dietary intake in these articles?

### 2.2 Information Sources

Databases searched included MEDLINE (all years; PubMed) [27], MEDLINE (1946 to April 2024; OvidSP) [28], CINAHL (all years; EBSCO) [29], and Scopus (all years; Elsevier) [30]. Grey literature was searched by using clinical trials registries (https://trialsearch.who.int, https://clinicaltrials.gov/) and pre‐print repositories (https://scholar.google.co.uk, https://www.biorxiv.org/, https://www.medrxiv.org/). A comprehensive search strategy was developed using keywords and indexed terms found in the titles and abstracts. The search strategy was tailored for each database to maximise coverage, and the final search was completed on 24 June 2024 (Table S2). Backward citation tracking was performed for all included articles. Only English‐language publications were included; no restrictions were applied to publication date.

### 2.3 Articles Selection

References were managed using Mendeley (v2.90, 2023) [31] and screened using Rayyan (https://www.rayyan.ai/). Two researchers (S.A. and R.G.) conducted the screening in Rayyan following a two‐stage blinded process: stage 1 title and abstract screening and stage 2 full‐text screening. Agreements were agreed upon by consensus with a third reviewer (M.L.).

### 2.4 Data Extraction and Synthesis

Data were extracted by one author (S.A.) using a modified JBI data charting tool [23] aligned with the review objectives. Extracted fields included study details (e.g., year, country, design), population characteristics (e.g., occupation, shift exposure, demographics), and measured variables (e.g., GI symptoms, diet, lifestyle factors). A 20% random sample was checked by another author (RG). Descriptive and narrative synthesis was conducted. Articles were grouped by study characteristics and classified by symptom assessment tools (e.g., Rome III, GSRS) and measurement types (e.g., Likert scales). Dietary variables were grouped by type (e.g., caffeine, alcohol). Age‐related data were reported using means and standard deviations (SD) or medians and interquartile ranges (IQRs) where applicable. In this review, self‐report questionnaires or tools refer to articles that utilised unnamed instruments for assessing GI and dietary behaviours. Additionally, the term ‘daytime’ refers to ‘day’ or ‘non‐shift work’, while ‘irregular shifts’ denotes non‐fixed shift schedules. For other terminologies, see Table S3.

## 3 Results

### 3.1 Study Inclusion

**Figure 1:**
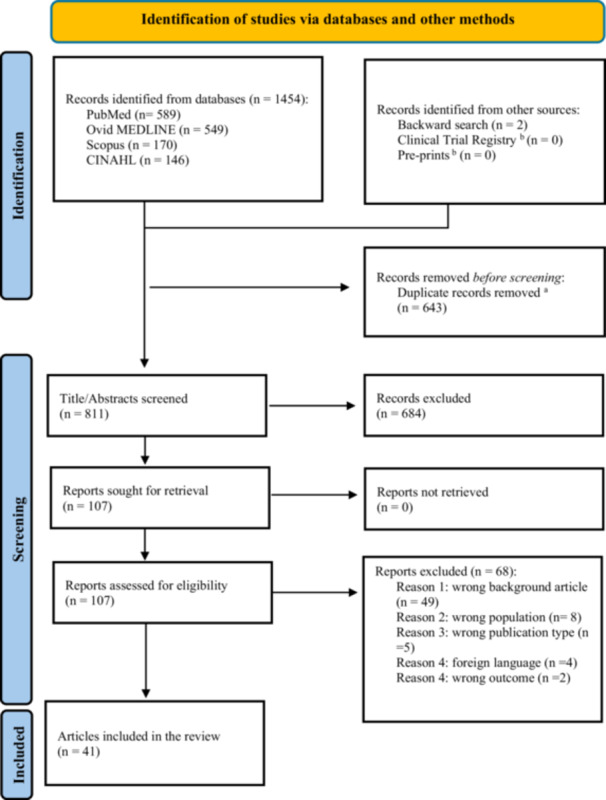
Preferred Reporting Items for Systematic Reviews and Meta‐Analyses (PRISMA) flow diagram of included studies in the systematic scoping review. *Source:* Page M. J., et al. *BMJ* 2021; 372: n71. doi: 10.1136/bmj.n71. a. Duplicates were removed by using Mendeley reference manager and Rayyan. All deduplications were verified manually. b. The Clinical Trial Registry included two registries: the WHO (International Clinical Trials Registry Platform Search Portal) and ClinicalTrials.gov, while the preprints were sourced from three repositories: Google Scholar, Medrxiv, and Biorxiv.

A total of 1454 articles were identified through database searches, with 811 remaining after duplicates were removed. After screening titles and abstracts, 107 articles underwent full‐text review, and 39 met the inclusion criteria. An additional 62 records were identified through grey literature and backward citation searching, yielding two more eligible articles. In total, 41 articles were included in the final synthesis (Figure 1).

### 3.2 Characteristics of the Included Articles

Most articles were observational, comprising cross‐sectional (*n* = 36, 87.8%) and prospective longitudinal (*n* = 3, 7.3%) design. The remaining two articles were randomised controlled trials (RCTs) (4.9%) (Table 1). The mean sample size was 918 (range: 10 to 17,529), with the two RCTs having the smallest samples (10 and 44 participants) [41, 42]. South Korea was the most represented country (*n* = 7, 17.0%) [12, 41, 42, 48, 52, 53, 68]. Definitions of shift work varied [12, 16, 33, 48, 52, 59, 66], including differences in shift length, start time, and extended on‐call duties lasting up to 32 h [44, 53]. Five articles (12.2%) used a within–person design [48, 49, 59, 60, 61].

**Table 1:** Characteristics of included articles.

Author, year, country	Design, occupation, setting	N (SW DW)	Mean age (years) (SD), Female (%)	Type of shift work	Comparator	Shift work definition*
Ahmed, 2017 [32] Egypt	Cross‐sectional, Nurse, Hospital	(112, 51)	34.7 (10.2), 93.3%	1—Rotating shift, 2—non‐fixed shift	Shift work vs. non‐shift work	NR
Alsadah 2021 [33] Saudi Arabia	Cross‐sectional, Nurse, Hospital	(194, 56)	SW: 28.2 (4.2). DW: 32.9 (5.3), 100%	2—DW, 2— Rotating shift	With stress vs. without stress	‘Nurses who worked between 6:00 AM and 7:00 AM over the last year were considered daytime workers. The shift work nurses worked rotating shifts, in which each nurse worked during the morning (7:00 AM–3:00 PM) for 1 month, during the evening (3:00 PM–11:00 PM.) for the next month, and during the night (11:00 PM–7:00 AM) following month’.
Burdelak W. et al., 2012 [34] Poland	Cross‐sectional, Nurse and midwife, NR	(354, 371)	Rotating: 48 (NR). DW: 50 (NR), 100%	1—Rotating night shift, 2— DW	Daytime vs. night rotating shift	NR
Caruso C et al., 2004 [35] USA	Cross‐sectional, Auto factory workers, Auto factory	(118, 225)	DW: 47 (5.7). Evening shift: 45.2 (7.0), 14.9%	Permanent day shifts (6:00–14:30) and permanent evening shifts (14:30–23:00)	Daytime vs. evening	‘DW 6:00–14:30 and for evening shift was 14:30–23:00’.
Cattani A. et al., 2022 [36] Brazil	Cross‐sectional, Nurse, Hospital	139^d^	42.6 (9.47), 86.3%	1—DW, 2— Night shift	Illness (physical, psychological, social) vs. sleep quality (good, poor)	‘DW (from 7 AM to 1 PM; and from 1 PM to 7 PM) and the night shift (from 7 PM to 7 PM)’.
Chan M., 2009 [37] Hong Kong	Cross‐sectional, Nurse, Hospital	163^c^	32.8 (6.9), 90.2%	Rotating shifts	Insufficient vs. sufficient sleep	‘Evening shift (15:00–23:00 h), Morning shift (7:00–15:00 h) and Night shift (23:00–7:00 h)’.
Ebrahim A. and Fredericks S., 2017 [38] Bahrain	Cross‐sectional, Nurse, Hospital	(49, 48)	Regular shift workers. 19.1 (2.86) Irregular shift workers. 21.9 (0.97), 75.3%	1—Morning shift only, 2—Irregular shift (morning, evening, and night)	Regular (1st year students) vs. irregular (4th year students) patterns of shiftwork	‘The shift periods for hospitals were defined as morning shifts (06:00 to 14:30) evening shifts (14:30 to 22:30) and night shifts (22:30 to 06:30)’.
Ede´ ll‐Gustafsson U. et al., 2002 [39] Sweden	Cross‐sectional, Casualty department, Hospital	(75, 81)	43.9 (10.4), 100.0%	1—DW, 2—Night shift, 3—Rotating shift	Years of shift experience	NR
Fido A. and Ghali A., 2007 [40] Kwait	Cross‐sectional, Blue‐collar workers, Oil Company (industrial setting)	(200, 200)	SW: 34.2 (7.3) Fixed shift workers: 34.5 (7.4), 0.0%	Variable schedule of 8‐h shifts. The shift schedules included morning, afternoon, night shifts, and free days in a specific pattern.	Years of shift work experience Fixed shifts (control group) vs. Shift workers (study group)	NR
Grant C. et al., 2017 [41] Australia	RCT, NR, Laboratory setting (stimulated shift work)	10^c^	24.7 (5.6), 0.0%	Stimulated night shift	Eating at night (control) vs. not eating at night condition (intervention)	Simulated shift work was described
Gupta C. et al., 2019 [42] Australia	RCT, NR, Laboratory setting (stimulated shift work)	44^c^	25.0 (2.9), 41.0%	Stimulated night shift	Meal at night, snack at night, no food	Simulated shift work was described
Hwang S. et al., 2022 [12] South Korea	Cross‐sectional, Nurse, Hospital	125^c^	28.0 (3.1), 100.0%	Rotating shift	Severe GI symptoms vs. without severe GI symptoms (mild)	‘Rotating shift nurses were those who worked in three shifts, including day, evening, and night, for more than one year and were still working in shifts’.
Ibrahim Nahla et al., 2016 [43] Saudi Arabia	Cross‐sectional, Nurse, Hospital	(152, 77) b	36.5 (NR), 92.1%	1—DW, 2—Alternating or night shift	IBS vs. non‐IBS	NR
Jaradat R. et al., 2020 [44] Jordan	Cross‐sectional, Post‐graduate resident physicians, Hospital	201^c^	27 (NR)^a^, 39.3%	Rotating shift	Rotating shift	‘The on‐call shift starts at 4 PM at the end of a normal working day until the next morning (8 AM) of another working day, in other words, the on‐call physician works continuously for 32 h’.
Jung H. and Lee B., 2016 [45] South Korea	Cross‐sectional, Nurse, Hospital	1431^c^	27.8 (NR), 98.0%	Rotating shift	Functional Dyspepsia vs. insomnia	NR
Katsifaraki M., 2019 [46] Norway	Prospective Longitudinal, Nurse, Hospital	679^c^	40.7 (11.13), 95.6%	Rotating shift	Night shift vs. morning shift	‘The type of shift was then categorised as follows. A shift starting between 05:00 and 12:00 was classified as morning shift, a shift starting between 12:01 and 18:00 as evening shift and a shift starting between 18:01 and 04:59 as night shift’.
Katsifaraki M., 2020 [47] Norway	Prospective Longitudinal, Nurse, Hospital	680^c^	NR, 90.6%	1—Morning shift, 2—Evening shift, 3—Night shift	Morning, night, quick return group	‘Shift type was categorised into three categories by shift starting time: morning shift (starting time 05:00–12:00), evening shift (starting time 12:01– 18:00) and night shift (starting time 18:01–04:59)’.
Ki J. et al., 2020 [48] South Korea	Cross‐sectional, Nurse, Hospital	500^c^	26.7 (4.2), 100.0%	1—DW, 2— Evening shift, 3—Night shift.	Within person (same individuals, novice and experienced nurses)	‘Shift work as a combination of daytime, evening, and night shifts’.
Knauth P. and Härmä M., 1992 [49] Germany	Cross‐sectional, Nurse, NR	147^c^	33.8 (NR), 100.0%	Morning shift and night shifts	Shift work tolerance (good, poor)	NR
Koh S. J. et al., 2014 [50] South Korea	Cross‐sectional, Nurse, Hospital	301^c^	FGIDs: 28.12 (4.88) Without FGIDs 29.48 (6.43), Subjects with FGIDs: 97.7%. without FGIDs: 96.2%.	1—Rotating shift, 2—DW shift	With vs. without FGIDs	NR
Korompeli A. et al., 2014 [51] Greek	Cross‐sectional, Nurse, Hospital	365^c^	NR, 86.5%	1—Morning shift, 2—Evening shift. 3—Night shift.	Irregular shift (including night shift) vs. permanently morning shift	NR
Lee S. et al., 2020 [52] South Korea	Cross‐sectional, Electronics manufacturing, Manufacturing company	(290, 964)	NR, 41.2%	1‐ DW 2‐ SW	Shift workers vs. day workers	‘The shift workers consisted of two groups with different work schedules, either 4 × 8 h (morning: 07.00– 15.00; afternoon: 15.00–23.00; night: 23.00–07.00) or 3 × 12 h (DW: 08.00–20.00; night: 20.00–08.00). As such, both groups of shift workers included night shift workers’.
Lim S. et al., 2017 [53] South Korea	Cross‐sectional, Doctor, Hospital	170^c^	FGIDs: 27.48 (2.70) Without FGIDs: 28.44 (3.23), 67.2%	24 h on call shift	With vs. without FGIDs (all with 24 h‐on‐call shifts)	‘A doctor with 24 h‐on‐call shifts is defined as doctors who experienced night on call duties, followed by routine daytime work. All doctors enroled in our study experienced a 24 h‐on‐call shift every other day, or once in every three days, for at least 6 months’.
Liu L. et al., 2014 [54] China	Cross‐sectional, Nurse, Hospital	340^c^	NR, 96.8%	NR, but includes night shift.	IBS vs. controls	NR
Ljevak I et al., 2021 [55] Bosnia	Cross‐sectional, Nurse, Hospital	157^c^	33.3 (8.033), 86.0%	1—DW shift (49% of participants) 2‐ SW: i.e., 12‐h day shift/24 h off/12‐h night shift/48 h off (51% of participants).	Daytime vs. shift workers	‘SW: 12‐h day shift/24 h off/12‐h night shift/48 h off. DW: consisting of seven working hours (i.e., from 7:30 AM to 2:30 PM)’.
Mendes S. and Martino M., 2012 [56] Brazil	Cross‐sectional, Nurse, Hospital	136^c^	33.1 (NR), 82.4%	Specific shifts (12‐h day)	Daytime vs. night shift workers	‘DW workers were from 7 AM to 7 PM, while the night shift workers worked from 7 PM to 7 AM of the next day’.
Nojkov B. et al., 2010 [57] USA	Cross‐sectional, Nurse, NR	(185, 214)	DW: 45.6 (9.3) Night shift workers: 41.5 (11.0) Rotating shift workers: 37.3 (10.8), 89.7%	1—Permanent DW shift workers. 2—Permanent night shift workers. 3—Rotating shift workers.	Daytime, night, rotating shifts	NR
Ottmann W et al., 1989 [58] Germany	Cross‐sectional, Police officer, NR	(2659, 1303)	NR, NR	1—Work shift, 2—DW shift.	Daytime workers and shift workers	NR
Pitsopoulos C. and Greenwood K., 2004 [59] Australia	Cross‐sectional, NR “various occupations”, NR “various industries”	28^c^	36.5 (9.9), 50.0%	Rotating shift	Comparison of shifts within the same individuals	‘Rotating shift‐cycle: Participants worked on a rotating shift cycle, which included at least two consecutive day shifts and at least two consecutive night shifts within a rotation period’.
Poole C. et al., 1992 [60] UK	Prospective Longitudinal, Factory workers, Factory setting	(138, 74)	DW: 36 (NR). SW: 33 (NR), 16.5%	1—Late shift, 2—Early shifts, 3—Night shifts	Fortnight about’, ‘3 shifts’, and day shifts.	NR
Rijk M. et al., 2021 [61] Netherlands	Cross‐sectional, Nurse, Hospital	118^c^	44.2 (30.1—51.6)^a^, 100.0%	Night shift	Night shift (all)	‘A night shift series was defined as a row of consecutive night shifts’.
Rogers A. et al., 2021 [62] USA	Cross‐sectional, Nurse, Hospital	(27, 24)	32.9 (10.0), 96.0%	1—DW, 2— Night shift	Night shift (including rotating as per the studies analysis) and day workers	NR
Roman P. et al., 2023 [16] Spain	Cross‐sectional, Nurse, Hospital	(221, 159)	24.8 (6.9), 92.9%	1—Rotating shift, 2—Fixed shift	Rotating vs. fixed shift workers	NR
Saberi H. et al., 2010 [63] Iran	Cross‐sectional, Nurse, Hospital	(133, 27)	NR, 73.2%	1—Rotating shifts, 2—Night shift, 3—DW shift.	Shift workers vs. day workers (morning only)	NR
Storz M. et al., 2022 [64] USA	Cross‐sectional, NR, NR	(458, 2007)	DW: 42.53 (NR). SW: 37.89 (NR), 45.9%	1—DW shift, 2—Shift workers (evening, night, and rotating shift workers).	Daytime vs. shift workers (regular evening shift, a regular night shift, or a rotating shift)	
Sveinsdóttir H., 2006 [65] Iceland	Cross‐sectional, Nurse, NR	(240, 154)	DW: 45.8 (8.1) DW/evening shift: 45.5 (9.2) DW/evening/night shift: 40.0 (9.3), 100.0%	1—DW shift only, 2— Rotating DW/evenings, 3—Rotating DW/evenings/nights	Daytime, Daytime/Evening, and Daytime/Evening/Night	NR
Xue J. et al., 2020 [66] China	Cross‐sectional, Workers (without specifying), Hospital	2027^c^	48.94 (4.16), 50.0%	Rotating shift	Rotating night shift work and nonrotating	‘Rotating night shift work was defined as at least three‐night shifts per month in addition to day shifts in that month’.
Yıldız F. and Esin M., 2009 [67] Turkey	Cross‐sectional, Nurse, Hospital	400 c	31.5 (7.1), 100.0%	1—DW, 2—Evening shift, 3—Night shift	Standard 8‐h shifts	‘Three shifts (8‐h shifts): 8:00 h–16:00 h (day shift), 16:00 h–0:00 h (evening shift), and 0:00 h–8:00 h (night shift)’.
Yun B. et al., 2022 [68] South Korea	Cross‐sectional, NR, Hospital	17,529	44.35 (8.75), 43.1%	Three shift and others	Normal GI or having constipation	‘The three‐shift system typically involves 8‐h shifts, with specific shift times: DW starts at 7 AM’.
Zhen Lu W. et al., 2006 [69] Singapore	Cross‐sectional, Nurse, NR,	(58, 60)	29 (NR), 100.0%	Rotating shift	Rotating shift nurses and regular day nurses	NR
Zhou H. et al., 2017 [70] China	Cross‐sectional, NR, Hospital	(215, 187)	31.4 (8.4), More than 98.0%	1—DW, 2— Rotating shift	Permanent daytime vs. rotating shifts	NR

Abbreviations: AM, Ante Meridiem (before midday); DW, daytime workers; FGIDs, functional gastrointestinal disorders; GI, gastrointestinal; IBS, irritable bowel syndrome; N, sample size; NR, not reported; PM, Post Meridiem (after midday); RCT, randomised control trial; SD, standard deviation; SW, shift workers; y, year.

^a^ Median and interquartile range (IQR).

^b^ Eight nurses didn't answer this question.

^c^ All were shift workers (SW).

^d^ 129 were working the night shift and 27 had another job.

### 3.3 Characteristics of Included Participants

Most articles focused on nurses *(n* = 27, 65.8%) [12, 16, 32, 33, 34, 36, 37, 38, 43, 45, 46, 47, 48, 49, 50, 51, 54, 55, 56, 57, 61, 62, 63, 65, 67, 69, 70], followed by factory workers [35, 60], doctors [53], police officers [58], and other occupations (Table 1). Ten articles (24.4%) had predominantly female participants [12, 33, 34, 39, 48, 49, 61, 65, 67, 69], two (4.9%) focused on males [40, 41], and one (2.4%) did not report gender [58]. Ethnicity was infrequently reported; only five articles (12.2%) included this information [35, 43, 54, 60, 64]. Similarly, participant health status was reported in six articles (14.6%) [32, 36, 37, 39, 43, 48]. The type of shift was primarily rotating shifts (*n* = 21, 51.2%) or night shifts (*n* = 19, 46.3%).

### 3.4 Gastrointestinal Symptoms

#### 3.4.1 Gastrointestinal Symptoms and Assessment Tools

A total of 23 self‐reported tools (56.0%) were used to assess GI symptoms across different articles, of which three were validated measures (Table 2). The Rome III criteria were the most frequently applied (*n* = 9, 22.0%) [38, 43, 45, 50, 53, 54, 57, 62, 70], including one article that utilised a Chinese version [54]. Other validated tools included the Gastrointestinal Symptom Question (GSQ; *n* = 4, 9.8%) [12, 33, 63, 69], and the Gastrointestinal Symptom Rating Scale (GSRS; *n* = 2, 4.9%) [16, 32]. Some articles used more general tools, such as the Standard Shiftwork Index (SSI) [34, 51, 59, 67], the Gut Reaction Scale [41], or a Visual Analogue Scale (VAS) with specific questions on upset stomach and bloating [42]. Others assessed the broader impact of GI symptoms on quality of life, employing measures like the Irritable Bowel Syndrome (IBS) Quality of Life Measure (IBS‐QOL) [57] and the Health and Well‐being Scale [55]. One article used a binary (yes/no) approach to assess health problems, including GI disorders such as gastric ulcers, diarrhoea, constipation, and stomachache [48]. One article did not specify the GI symptoms measured [65] (Table 2).

**Table 2:** Gastrointestinal Symptoms and Assessment Tools.

Assessment tool/diagnostic criteria	Self‐reported	Description/measurement	Validated (yes/no/NR)	Reference(s)
Rome III	Yes	Assessed via structured or self‐reported questionnaires, often incorporating symptom duration, frequency, and subtyping of FGIDs; several studies used Likert‐type scales to rate symptom severity or prevalence.	Yes	[38, 43, 44, 50, 53, 54, 62, 70]
Non‐standardised self‐reporting questionnaire	Yes	Self‐reporting questionnaire: 3‐point Likert scale, dichotomised into one or more times/week and not once/week, ‘Yes or No’ answer options, (Often or continuously; sometimes; rarely or never), Answers as %: seldom or always, (never, often seldom), 5‐point Likert scale,	NR	[35, 36, 40, 44, 48, 49, 56, 58, 60, 68]
Gastrointestinal Symptom Rating Scale (GSRS)	Yes	4‐point or 7‐point Likert scale	Yes	[16, 32]
Standard Shiftwork Index (SSI)	Yes	3‐point Likert scale, Part of the ‘Physical Health Questionnaire’	NR	[34, 51, 59, 67]^d^
Strain and Symptoms Questionnaire (SSQ)	Yes	5‐point Likert scale	NR	[37, 39]
Gastrointestinal Symptoms Questionnaire	Yes	Thirty‐two GI symptoms were covered over the previous 4 weeks (last month). Likert scale (4‐point), 7‐point Likert scale, 7‐point Likert scale, 4‐point Likert scale.	Yes	[12, 33, 67, 69]
Self‐reported pain diary	Yes	4‐point Likert scale	NR	[46, 47]
Gut Reaction Scale	Yes	Gut reaction 9‐point Likert scale	NR	[41]
Visual Analogue Scale (VAS)	Yes	Stomach upset (No stomach upset to extremely upset stomach) Bloating ‘I don't feel bloated to I feel very bloated’	NR	[42]
IBS Severity Scoring System (IBS‐SSS)^a^	Yes	A scale from 0 to 100	NR	[43]
The Korean Occupational Stress Scale (KOSS)	Yes	Subjective measures (GI symptoms questions)	NR	[52]
A validated Korean version of Bowel Disease Questionnaire (BDQ‐K)^a^	Yes	Diagnostic classification	NR	[53]
IBS Severity questionnaire^a^	Yes	Diagnostic classification. 100‐point scale	NR	[54]
Health and Wellbeing scale	Yes	4‐point Likert scale	NR	[55]
Irritable Bowel Syndrome‐Quality of Life Measure (IBS‐QOL)^a^	Yes	34‐item survey	NR	[57]
1‐ Bowel Health Questionnaire (BHQ) 2‐ Faecal Incontinence Severity Index (FISI) 3‐ The Bristol Stool Form Scale (BSFS)	Yes	Incorporated stool measure	NR	[64]
Gastrointestinal symptom scale	Yes	GI symptoms based on three symptoms	NR	[65]
Frequency scale for symptoms of GERD (FSSG)	Yes	5‐point Likert scale	NR	[66]
The Irritable Bowel Syndrome Symptoms Evaluation Questionnaire b	Yes	4‐point Likert scale	NR	[69]

Abbreviations: FBD, functional bowel disorders; FD, functional dyspepsia; FGIDs, functional gastrointestinal disorders; GERD, gastro‐oesophageal reflux; GI, gastrointestinal; IBS, irritable bowel syndrome; NR, not reported; QOL, quality of life.

^a^ Used with Rome III criteria.

^b^ Used with Gastrointestinal Symptom Questionnaire.

^c^ Chinese version.

^d^ Used the Physical Health Questionnaire included in the SSI.

#### 3.4.2 Gastrointestinal Symptoms and Shift Work

##### 3.4.2.1 Shift Work Type and Comparison Groups

Shift workers were most frequently compared to daytime workers (*n* = 15, 36.6%) [33, 41, 42, 44, 45, 48, 49, 50, 52, 53, 59, 60, 61, 66, 68]. Other comparisons included shift timing (*n* = 4, 9.8%) [46, 47, 60, 61], years of shift work experience (*n *= 2, 4.9%) [39, 48], presence of clinical GI conditions such as IBS (*n* = 2, 4.9%) [43, 54] and functional dyspepsia (*n *= 1, 2.4%) [45]. Some articles used sleep‐related outcomes (*n *= 2, 4.9%) [36, 37], or stress levels (*n* = 1, 2.4%) [33] as the basis for comparison (Table 1).

##### 3.4.2.2 Gastrointestinal Symptoms and Shift Work

More than half of the articles (*n* = 23, 56.0%) showed significant associations between various GI symptoms and shift work. These included 21 cross‐sectional (51.2%) [16, 32, 33, 34, 35, 38, 40, 43, 51, 52, 54, 55, 56, 57, 58, 59, 61, 63, 66, 67, 69] and two prospective longitudinal (4.8%) [46, 47] designs.

Among these, eleven articles (26.8%) [32, 33, 35, 51, 52, 55, 58, 61, 63, 65, 67] reported on general GI symptoms (e.g., digestive problems) in shift workers, with eight (19.8%) identifying a positive association [32, 33, 52, 55, 58, 59, 61, 63]. One article assessed mean (SD) GSRS scores and found that shift workers had higher scores compared with daytime workers: 0.73 ± 0.33 SD versus 0.59 ± 0.42 SD [32]. Similar trends were reported in other articles comparing shift workers with daytime workers [33, 52, 55, 58, 63], night shift workers with daytime workers [59], and within the night shift workers group [61].

Three articles (7.3%) [16, 38, 40] reported a higher frequency of constipation among rotating or irregular shift workers. One article found significantly increased odds of constipation (OR = 1.904, 95% CI: 1.200–3.021) compared to regular shift workers [38].

Night shift workers were more likely to report bloating and flatulence. One article indicated that 25.0% of night shift workers ‘always’ experienced these symptoms compared to 8.3% of daytime workers [56].

Findings on IBS prevalence among shift workers were mixed. Three articles (7.3%) found higher rates of IBS among rotating or night shift workers compared to daytime workers [43, 54, 57], with one reporting an odds ratio of 2.79 (95% CI: 1.32–5.90) [43]. Conversely, one article found that nurses working five to seven night shifts per month had significantly lower odds of IBS compared to those working fewer night shifts (OR = 0.13; 95% CI: 0.02–0.80) [34]. Two articles reported no significant association between IBS and shift work, one comparing rotating versus daytime shifts [50], and the other comparing night versus daytime shifts [61].

Abdominal pain appeared to be more prevalent among night shift workers. One article (2.4%) reported that nurses had over three times the odds of experiencing abdominal pain after night shifts compared to morning shifts (adjusted OR = 3.17, 95% CI: 1.80–5.60) [47]. Another article reported higher abdominal pain scores in night shift workers (OR = 1.63, 95% CI: 1.36–1.94), while evening shift workers had lower scores (OR = 0.73, 95% CI: 0.62–0.86) [46].

One article (2.4%) reported that rotating night shift work was independently associated with gastro‐oesophageal reflux (GORD) symptoms compared to daytime workers (OR = 3.66, 95% CI: 2.52–5.40) [66]. Symptom severity varied by shift pattern. Evening and rotating shift workers consistently reported more severe GI symptoms than daytime‐only workers [35, 65, 69].

### 3.5 Dietary Behaviours

A total of 12 articles (29.3%) assessed dietary behaviours [12, 32, 40, 44, 45, 54, 60, 61, 64, 66, 67, 70], using nine different tools, of which five were validated [12, 40, 61, 64, 67, 70]. All dietary data were self‐reported and collected through various measures, including study‐specific dietary questionnaires (*n* = 5, 12.2%) [32, 40, 44, 54, 60]; 24‐h dietary recalls (*n* = 2, 4.9%) [61, 64], one conducted over 3 days using a validated app [61] and the other primarily over a single day [64]; the Dietary Habit questionnaire (*n* = 1, 2.4%) [12]; the Health‐related Characteristics questionnaire (*n* = 1, 2.4%) [45]; the Standard Shift Work Index (SSI) (*n* = 1, 2.4%) [67]; and a 47‐item questionnaire on IBS (*n* = 1, 2.4%) [70]. One article did not specify the dietary assessment tool used [66] (Table 3).

**Table 3:** Dietary behaviours and assessment tools.

Dietary assessment tool	Validated (yes/no/NR)	Nutrients or factors measured	Recall Period	Contextual relevance to shift work	Reference(s)
Study‐specific dietary questionnaire	No	Coffee and tea consumption	Habitual intake	General	[32]
Study‐specific dietary questionnaire	Yes	Coffee and tea consumption	Habitual intake	General	[40]
Dietary Habit Questionnaire	Yes	1—Healthy and unhealthy dietary habits,^a^ 2—Alcohol and caffeine consumption per week	Habitual intake	General	[12]
Study‐specific dietary questionnaire	No	Caffeine consumption	Habitual intake	General	[44]
Health‐related characteristics questionnaire	No	Meal regularity and alcohol intake	Habitual intake	General	[45]
Study‐specific dietary questionnaire	No	Alcohol consumption	Habitual intake	General	[54]
Study‐specific dietary questionnaire	NR	Alcohol consumption	Habitual intake	General	[58]
24‐h dietary recalls	Yes	Energy intake, caffeine consumption, and eating frequency	Three 24‐h dietary recalls	Shift‐specific (each 24‐h recall was completed after the first night shift). Participants recorded everything they consumed during 24‐h, from the evening meal before the night shift until the evening meal after the night shift. Meal timing was recorded.	[60]
24‐h dietary recalls	Yes	Macro and micronutrient intake. Saturated, monounsaturated, and polyunsaturated fat intake, fibre, alcohol, and caffeine consumption.	One‐day 24‐h dietary recalls	General	[64]
NR	NR	Alcohol consumption	NR	General	[66]
Physical Health Questionnaire (Part of the Standard Shiftwork Index)	Yes	Caffeine consumption and use of vitamins	Habitual intake	General	[67]
47‐item questionnaire on IBS	Yes	Food habits (high protein food, spicy food, fried food, starchy food, caffeine consumption, and fibre‐rich foods	Habitual intake	General	[70]

Abbreviation: IBS, irritable bowel syndrome.

^a^ Assessed the amount of consumption only, not linking the dietary findings to GI symptoms.

^b^ Assessed the amount of consumption between shift status (daytime vs. shift workers).

^c^ Healthy dietary habits were measured based on the frequency of intake of grains, proteins, fruits, vegetables, and dairy foods. Unhealthy dietary habits were assessed by analysing the intake frequency of fatty foods, instant foods, fast foods, and night meals.

^d^ Seven participants completed 1 day 24‐h recall, 14 completed 2 days 24‐h recall, and 97 completed all 3 days 24‐h recall. Eating frequency was defined as eating occasion in which all foods and drinks consumed in one time was considered one eating occasion, a new eating occasion if it was consumed in at least 15 min apart.

*Rated on a scale from 1 (*rare intake*) to 5 (*frequent intake*). Non‐ quantitative Food Frequency Questionnaire (FFQ).

The dietary behaviours explored seven distinct domains, including caffeine intake (*n* = 8, 19.5%), alcohol consumption (*n* = 6, 14.6%), fibre intake (*n* = 3, 7.3%) [12, 64, 70], meal regularity (*n* = 1, 2.4%) [45], vitamin use (*n* = 1, 2.4%) [67], food habits including the consumption of high‐protein, spicy, fried, and starchy foods (*n* = 1, 2.4%) [70], and water intake (*n* = 1, 2.4%) [64]. Four of the twelve articles reported only descriptive dietary intake data without comparing shift types [40, 44, 60, 64]; these included two on caffeine consumption, one on alcohol intake, and one assessing fibre, caffeine, and water intake (Table 3).

#### 3.5.1 Caffeine Intake

Eight articles (19.5%) assessed caffeine intake [12, 32, 40, 44, 61, 64, 67, 70], with four examining associations with GI symptoms [12, 32, 61, 70] (Table 4). Caffeine sources were identified as primarily coffee and tea in two articles [32, 40].

**Table 4:** Dietary behaviours and GI symptoms.

Study design	Dietary factor	Reported GI symptom	Comparison	Direction of association (+/−)	Reference(s)
Cross‐sectional	Coffee and tea consumption	Abdominal pain, reflux, diarrhoea, indigestion, GSRS score	Shift vs.daytime workers	+ (Increased risk)	[32]
Cross‐sectional	Caffeine intake ( > 1 cup/day), lower protein and vegetable intake, and alcohol consumption	Higher total GI symptom scores, with more caffeine and lower protein/vegetable intake in nurses with severe GI symptoms	Severe GI symptoms vs. without severe GI symptoms in rotating shift workers	+ (Increased risk for caffeine & low protein/vegetables); no significant association for alcohol	[12]
Cross‐sectional	Meal regularity, alcohol consumption	FD	Functional Dyspepsia vs. insomnia in rotating shift workers	+ (Increased risk)	[45]
Cross‐sectional	Alcohol consumption	IBS	IBS vs. controls in night shift workers	+ (Increased risk)	[54]
Cross‐sectional	Caffeine consumption	Heartburn and bloating, gastric pain, constipation, diarrhoea, or growling intestines.	Night shift	+ (Increased risk for heartburn and bloating)	[61]
Cross‐sectional	Alcohol consumption	No association with GERD symptoms after adjusting for confounders	Rotating night shift work vs. daytime workers	No significant association	[66]
Cross‐sectional	Use of vitamins a	Higher GISs subscale score	Different subgroups of shift workers (all shift workers)	+ (Increased risk)	[67]
Cross‐sectional	Food habits (high protein food, spicy food, fried food, starchy food, caffeine consumption, and fibre‐rich foods	No significant association between these habits and functional GI disorders	Permanent daytime vs. rotating shifts	No significant association	[70]

Abbreviations: FD, Functional dyspepsia; GERD, gastro‐oesophageal reflex disease; GI, gastrointestinal; GIS, gastrointestinal symptom; GSRS, Gastrointestinal Symptom Rating Scale; IBS, irritable bowel syndrome; NR, not reported.

^a^ No information provided about the specific types or forms of vitamins taken.

One article [32] reported elevated symptoms among participants consuming more than three cups of caffeine‐based drinks daily versus those consuming fewer than three cups. Another article [12] found a significant association for intake exceeding one cup per day (Cohen's *d* = 0.37) when comparing participants with severe GI symptoms to those without. In one cohort of night shift nurses, increased caffeine intake correlated with greater reports of heartburn and bloating, with a median intake of 37.3 mg/day (IQR: 0.0–100.5) [61].

Two articles reported nonsignificant results regarding caffeine intake, with one finding no association between caffeine consumption and GI symptoms [70], and another reporting no significant differences in caffeine intake across clinical areas worked (surgical, ICU, medical, operating rooms) [67].

#### 3.5.2 Alcohol Consumption

Six articles (14.6%) assessed alcohol intake [12, 45, 54, 60, 64, 66], with four linking it to GI symptoms [12, 45, 54, 66]. Two articles (4.9%) found potential associations with functional GI disorders (FGIDs) [45, 54]. One article linked heavy drinking (≥ 2x/week, ≥ 5 drinks for women, ≥ 7 for men) to functional dyspepsia [45], and another found increased IBS risk based on Yes/No alcohol use [54]. Another article noted a nonsignificant trend toward higher GI symptoms with more frequent alcohol use (≥ 3 vs. < 3 times/week) in rotating shift workers [12]. One article found no link between alcohol intake and GORD in night versus daytime shift workers [66]. None of the included articles examined dose–response relationships between alcohol intake and GI symptoms (Table S4).

#### 3.5.3 Fibre Intake

Three articles (7.3%) assessed fibre intake [12, 64, 70], of which two (4.9%) [12, 70] evaluated associations with GI symptoms. One article reported that low vegetable intake, indicated by responses near ‘rarely’, was associated with increased GI symptoms among rotating shift workers, regardless of symptom severity [12]. Another article found no significant association between Fibre‐rich food consumption, categorised as ‘usually/often’ versus ‘occasional/none’, and FGIDs among nurses working rotating versus permanent daytime shifts [70].

#### 3.5.4 Other Dietary Factors

Three articles (7.3%) assessed other dietary components [45, 67, 70]. One article assessed meal regularity on a 5‐point scale (1 = *very irregular* to 5 = *very regular*) and found that workers with higher‐than‐average meal regularity scores ( > 2.01) had significantly lower odds of experiencing functional dyspepsia compared to those with lower scores (OR = 0.67; 95% CI: 0.50–0.91) [45]. Another article found that vitamin use, compared to non‐use, was associated with higher GI symptom scores, including nausea, heartburn, bloating, constipation, and diarrhoea [67]. A third article categorised intake of specific food types (e.g., protein, spicy food) as ‘usually/often’ versus ‘occasional/none’ and found no consistent association between higher intake of high‐protein or spicy foods and GI symptoms [70].

### 3.6 Other Influencing Factors

Sleep, psychological, occupational, and demographic factors were frequently associated with GI symptoms among shift workers. Poor sleep quality [36, 43, 70], insufficient sleep [37], and sleep impairment [53] were consistently linked to a higher GI symptom burden.

Psychological stressors also emerged as important modifiers. Work‐related stress was associated with higher odds of functional dyspepsia compared to lower stress levels (OR = 1.37; 95% CI: 1.09–1.73) [45], while psychosocial distress was associated with a greater prevalence of both IBS and functional dyspepsia [50, 53]. Occupational stress, relative to lower or no reported stress, was linked to a broader range of GI symptoms, including gastritis [52], abdominal pain, heartburn, bloating, and nausea [33]. Work schedule characteristics were also influential. One article found that factory workers who transitioned from the ‘fortnight about’ pattern to a three‐shift schedule had a higher prevalence of indigestion [60]. Additionally, longer duration of shift work was associated with more frequent gastric symptoms, particularly among those dissatisfied with their working hours compared to those satisfied [39, 55].

Demographic factors, such as age, also appeared to influence outcomes. The prevalence of IBS was significantly higher among nurses aged ≤ 30 years (19.2%) compared to those over 30 years (8.5%) [43]. Similarly, nurses aged 20–30 years had significantly higher odds of reporting GI symptoms compared to those aged 31–50 years (OR = 13.90; 95% CI: 2.04–94.44) [67]. Another article found that age modified shift work and symptoms relationships. The prevalence of indigestion and appetite disturbances together decreased across age groups [19, 20, 21, 22, 23, 24, 25, 26, 27, 28, 29, 30, 31, 32, 33, 34, 36, 37, 38, 41, 42, 43, 44, 45, 46, 47, 48, 49, 50, 51, 52, 53, 54, 55, 56, 57, 59, 60, 61, 66, 68], with a significant interaction observed for overall GI symptoms [58].

## 4 Discussion

Shift workers frequently report concerns about the impact of irregular working hours on their dietary habits, GI health, and overall well‐being [71]. To our knowledge, this is the first systematic scoping review to map the evidence on GI symptoms and dietary behaviours in shift‐working populations.

General GI symptoms such as digestive discomfort, IBS, and constipation were frequently observed and appeared to be positively associated with shift work, aligning with existing literature on circadian rhythm disruption and GI health [46, 47]. Nonetheless, a few articles in this review found no significant associations [50, 61], and one reported a lower likelihood of IBS among shift workers compared to daytime workers [34]. This contradictory finding may reflect the ‘Healthy Worker Effect’, where healthier individuals remain in demanding shift roles, potentially skewing prevalence. Importantly, this article did not employ unique assessment tools for GI symptoms, nor did it include non‐shift workers, suggesting that other contextual factors, such as unmeasured health behaviours or workplace support, may have contributed.

This review consistently identified associations between poor sleep quality, irregular sleep [36, 37, 43, 53, 70], and psychological stress [33, 45, 50, 52, 53] with GI symptoms in shift workers. These findings suggest plausible physiological pathways involving circadian disruption, sleep impairment, and stress‐related neuroendocrine mechanisms. Sleep disturbances may alter gut motility and disrupt the gut–brain axis [53], while occupational stress may increase visceral sensitivity and inflammation [45]. Work schedule and shift duration also appear to influence symptom burden, potentially through cumulative circadian misalignment and stress [39, 55, 60]. Age‐related differences [43, 58, 67] indicate variability in susceptibility. Only one article used a validated stress model to assess its direct relationship with GI [33], highlighting the need for standardised stress assessment. Interestingly, one article found lower prevalence of indigestion and appetite disturbances in older adults [58], possibly due to dietary changes. However, the absence of dietary assessment weakens the reliability of this interpretation.

The literature was dominated by a cross‐sectional study design, limiting temporal insights. Only a few articles employed within‐person [48, 49, 59, 60, 61] and longitudinal [46, 47], approaches, which are better suited to assess dose–response relationships. Most RCTs were in simulated rather than real‐world settings [41, 42].

There was also substantial variability in how shift work was defined, ranging from timing and duration [12, 16, 33, 48, 52, 59, 66] to the inclusion of extended on‐call duties [44, 53], which may increase the risk of exposure misclassification and contribute to heterogeneity in findings [72].

GI symptoms were assessed exclusively through self‐report, with no clinical confirmation, raising concerns about recall and reporting bias, especially in retrospective formats [73, 74]. Although several articles employed validated measures such as the Rome III criteria [38, 43, 45, 50, 53, 54, 57, 62, 70] or the GSRS [16, 32], others relied on non‐standardised or inadequately described tools. One article did not specify which symptoms were measured [65], and another after the publication of Rome IV, further highlighting inconsistency [62]. The Rome IV criteria are now widely endorsed for diagnosing FGID in the United Kingdom [75, 76, 77] and globally [76, 78], with strong cross‐cultural validity demonstrated in a study of over 54,000 participants from 26 countries [79].

Despite the well‐established influence of diet on GI symptoms in shift workers [80, 81], fewer than one‐third of the included articles assessed dietary behaviours, all relying on self‐report, similar to those used for GI symptoms assessment, which limits interpretability.

Caffeine and alcohol intake were the most frequently examined components, though findings were inconsistent. Some articles found higher caffeine intake associated with increased GI symptoms [12, 32, 61], while one article reported no significant association [70]. Alcohol findings were similarly mixed [45, 54], likely due to unclear serving sizes, frequency, and missing context like timing. Only two articles specified caffeine sources (coffee or tea) [32, 40], and one omitted the alcohol assessment tool [66]. Grouping all caffeine‐containing products together may obscure the effects of additional ingredients, such as sugar or additives, on GI symptoms [82].

Fibre‐related findings were also inconsistent. One article linked low vegetable intake to higher symptom burden [12], while another did not specify fibre type [70]. None of the articles distinguished between soluble and insoluble fibre, limiting the specificity of dietary analysis. Limited access to fibre‐rich foods during night shifts may partly explain the consistently low intake observed among shift worker [83, 84].

Meal timing and regularity, key factors in circadian and digestive regulation, were rarely addressed. One article found that regular eating patterns were associated with reduced odds of functional dyspepsia [45], while another did not define regularity [61].

Other dietary factors were minimally explored. One article linked vitamin use to higher GI symptom scores [67], although it did not specify the types of supplements used. This is noteworthy, as nutrients such as iron and magnesium are known to cause GI side effects [85, 86], suggesting that specific compounds may underlie this association. Additionally, over‐the‐counter medicines may also contribute to GI symptoms and management, although their use was not identified in the articles. Hydration and general dietary habits (e.g., spicy, fried, or starchy foods) were also rarely assessed. One article found no difference in water intake between groups and did not examine its association with symptoms [64].

Accurate dietary assessment in shift workers remains challenging due to irregular schedules, fatigue, and limited food availability during night shifts, which may increase recall bias [87, 88]. Despite evidence supporting real‐time, time‐stamped dietary logging for this population [89, 90], only one article used a validated digital dietary app [61]. The use of tailored digital tools may enhance dietary data quality in future research.

Substantial heterogeneity in shift work definitions, participant populations, and outcome measures was observed, complicating synthesis and limiting comparability. For instance, one article [58] reported a combined outcome of indigestion and appetite disturbances, which does not align with standard GI symptom definitions, thereby complicating cross‐study comparisons.

Most articles focused on nurses, particularly in South Korea, a population that may experience relatively structured shift patterns and greater access to food and rest facilities [91]. Additionally, the overrepresentation of female participants with formal healthcare training raises concerns about occupational and gender representation in the literature. The scarcity of UK‐based research [60] further highlights the need for broader, more inclusive research across regions and professions.

This scoping review provides the first comprehensive synthesis of GI symptoms and dietary behaviours in shift workers. Strengths include a broad search strategy and rigorous data extraction. Limitations include the exclusion of non‐English articles and lack of formal quality appraisal due to the scoping nature. Despite these limitations, this review offers a valuable foundation for guiding future research.

## 5 Conclusion

This scoping review mapped evidence on GI symptoms and dietary behaviours in shift‐working populations, identifying common findings and methodological gaps. Several articles reported associations between shift work and general GI symptoms, IBS, and constipation. Despite diet modification being a first‐line management strategy for GI symptoms, few articles explored the role of diet in symptom development or progression. When diet was assessed, the focus was often limited to single components like caffeine and alcohol, frequently without consideration of timing or contextual factors.

Key methodological limitations included inconsistent definitions of shift work, limited use of validated dietary and symptom assessment tools, and underrepresentation of diverse occupational groups. Future research should address these gaps by employing shift‐adapted, time‐specific tools and longitudinal designs. These approaches will enhance understanding of GI health in shift workers and support the development of targeted workplace health interventions (Box 1).

LOI policies focus on the mandated language teachers should use when teaching students in the classroom. This review looked at whether MT‐based LOI and language transition policies facilitated reading and biliteracy and multilingual literacy outcomes for students and whether these policies have different effects on skill development by language group.

Box 1: **Recommendations for future research.**

1

- Employ within‐person and longitudinal study designs to better capture the dynamic impact of shift work on GI symptoms and dietary behaviours.
- Use validated instruments and objective, real‐time dietary assessment tools, such as food diaries or mobile apps with time‐stamped entries, that align with GI symptomatology and account for meal timing.
- Investigate understudied dietary factors, including meal timing, hydration, and the use of vitamins or supplements, which may play important roles in GI health but remain inadequately assessed.
- Consider the impact of over‐the‐counter medicines use when evaluating GI symptoms, as these may act as potential confounders.
- Incorporate validated measures of occupational stress and sleep quality into study protocols to evaluate their interacting roles as potential mediators in the relationship between shift work and GI symptoms.
- Expand study populations beyond healthcare professionals to include a wider range of occupations and geographic regions with diverse shift work structures.
- Clearly define shift work schedules (e.g., night, rotating, on‐call) in study protocols and reporting to enhance comparability and interpretation.
- Develop and validate dietary assessment tools specifically designed for shift‐working populations, accounting for irregular eating patterns, disrupted circadian rhythms, and access constraints.

## Author Contributions

**Seham H. Alyami:** conceptualisation, methodology, investigation, writing – original draft, writing – review and editing. **Miranda C. E. Lomer:** conceptualisation, methodology, supervision, writing – review and editing. **Rachel Gibson:** conceptualisation, methodology, supervision, investigation, writing – review and editing.

## Conflicts of Interest

Rachel Gibson holds unpaid roles as Research Leads for the British Dietetic Association Work Ready programme and HercuWise Ltd. Miranda C. E. Lomer leads post registration courses for dietitians on the dietary management of irritable bowel syndrome.

## 1 Transparency Declaration

The authors confirm that this manuscript represents a complete, accurate, and transparent account of the study. The work has been conducted and reported in accordance with the PRISMA‐ScR guidelines. The protocol is registered at the Open Science Framework, https://doi.org/10.17605/OSF.IO/ZYN27.

## 2 Peer Review

The peer review history for this article is available at https://www.webofscience.com/api/gateway/wos/peer-review/10.1111/jhn.70114.

## Supporting information

Supporting materials are available online in the Supporting Information section.

**Table S1:** Inclusion and exclusion criteria. **Table S2:** Detailed search strategy. **Table S3:** Terminologies. **Table S4:** Thresholds and dose‐response evidence for caffeine and alcohol consumption and GI symptoms.

## Data Availability

This scoping review did not generate or analyse any primary data; therefore, data sharing is not applicable.
