# Supplementary material for: Gastrointestinal Symptoms and the Assessment of Diet in Shift Workers: A Systematic Scoping Review
Source: J Hum Nutr Diet. 2025 Aug 28;38(5):e70114. doi: 10.1111/jhn.70114 (PMC12394927; doi:10.1111/jhn.70114)
Supplement: Supplementary file 1 — Table S1: Inclusion and exclusion criteria. Table S2: Detailed search strategy. Table S3: Terminologies. Table S4: Thresholds and dose‐response evidence for caffeine and alcohol consumption and GI symptoms. [file JHN-38-0-s001.docx]

**Gastrointestinal symptoms and the assessment of diet in shift workers: A systematic scoping review**

**Supplementary Information**

**Table S1** Inclusion and Exclusion Criteria

| Inclusion Criteria | Exclusion Criteria |
| --- | --- |
| Humans | Pregnancy or breast feeding |
| Shift workers – free living | Articles not written in English |
| Articles using simulated shift work schedule | Articles conducted on animals |
| Self-report or clinical measures of at least one GI symptom. | Articles that featured gastrointestinal (GI) disease |
| If the article included dietary assessment, it can be direct or indirect assessment. ^a^ | Articles which involve the occupation (e.g. nurses); however, don’t mention shift work exposure. |

 a. Direct dietary assessment tools such as self-reported intake (e.g., Food Frequency Questionnaires, 24-hour dietary recalls, food records/diaries), observed intake (e.g., weighed food records, direct meal tracking), biomarkers of dietary intake (e.g., blood nutrient levels, urinary sodium for salt intake).

**Table S2** Detailed Search Strategy

**Databases Search**

**PubMed:**

1- (“Shift schedule*” OR “Shift Work”  OR Shiftwork OR “Night Shift” OR “Night Duty” OR “Overnight Work”  OR “Evening shift”  OR “Evening work”  OR “Late-shift” OR “Graveyard shift”  OR “Third shift”   OR “Rotating Shift”  OR “Rotational shift” OR “Afternoon Shift” OR “Afternoon work”  OR “Morning Shift” OR “Morning work”  OR “Night work” OR “Non-day Work” OR "Alternative shift" OR "irregular work schedule"  OR "Work Schedule Tolerance"[Mesh] OR “Simulated shift work” OR “Circadian Rhythm*” OR “Day Shift” OR “Irregular shift” OR “Extended shift” OR “Circadian rhythm* disruption*”)

2- ("Gastrointestinal symptom*" OR "Abdominal pain" OR Nausea OR Vomiting OR Diarrhea OR Diarrhoea OR Constipation OR Indigestion OR Heartburn OR Bloating OR Melena OR Distension OR Dyspepsia OR Flatulence OR "Bowel change*" OR Regurgitation OR "Acid reflux" OR Emesis OR "Irritable bowel" OR "Functional gut" OR "Functional gastrointestinal" OR "Functional bowel" OR "gut-brain interaction" OR Borborygmi OR Gurgling)

3- 1 AND 2

**Ovid Medline:**

1. (Shift adj3 schedule*).mp. OR Shift Work.mp. OR Shiftwork.mp. OR Night Shift.mp. OR Night Duty.mp. OR Overnight Work.mp. OR Evening shift.mp. OR Evening work.mp. OR Late-shift.mp. OR Graveyard shift.mp. OR Third shift.mp. OR Rotating Shift.mp. OR Rotat* shift.mp. OR Occupational Night Worker*.mp. OR Afternoon Shift.mp. OR Afternoon work.mp. OR Morning Shift.mp. OR Morning work.mp. OR Night work.mp. OR Non-day Work.mp. OR Alternating Work.mp. OR "irregular work schedule".mp. OR Disturbed work schedule.mp. OR Simulated shift work.mp. OR Circadian Rhythm*.mp. OR Day Shift.mp. OR Irregular shift.mp. OR Extended shift.mp. OR Work Schedule Tolerance.mp. OR Circadian rhythm* disruption*.mp.
2. (Gastrointestinal adj3 symptom*).mp. OR Abdominal pain.mp. OR Nausea.mp. OR Vomiting.mp. OR Diarrhea.mp. OR Diarrhoea.mp. OR Constipation.mp. OR Indigestion.mp. OR Heartburn.mp. OR Bloating.mp. OR Melena.mp. OR Distension.mp. OR Dyspepsia.mp. OR Flatulence.mp. OR Bowel change*.mp. OR Regurgitation.mp. OR Acid reflux.mp. OR Emesis.mp. OR Irritable bowel.mp. OR Functional gut.mp. OR Functional gastrointestinal.mp. OR Functional bowel.mp. OR Disorders of gut-brain interaction.mp. OR Borborygmi.mp. OR Gurgling.mp.
3. 1 AND 2

*mp= [mp=title, book title, abstract, original title, name of substance word, subject heading word, floating sub-heading word, keyword heading word, organism supplementary concept word, protocol supplementary concept word, rare disease supplementary concept word, unique identifier, synonyms, population supplementary concept word, anatomy supplementary concept word]

**CINAHL**

1. (“Shift schedule*” OR “Shift Work”  OR Shiftwork OR “Night Shift” OR “Night Duty” OR “Overnight Work”  OR “Evening shift”  OR “Evening work”  OR “Late-shift” OR “Graveyard shift”  OR “Third shift”   OR “Rotating Shift”  OR “Rotational shift” OR “Afternoon Shift” OR “Afternoon work”  OR “Morning Shift” OR “Morning work”  OR “Night work” OR “Non-day Work” OR "Alternative shift" OR "irregular work schedule"  OR "Work Schedule Tolerance"[Mesh] OR “Simulated shift work” OR “Circadian Rhythm*” OR “Day Shift” OR “Irregular shift” OR “Extended shift” OR “Circadian rhythm* disruption*”)
2. ("Gastrointestinal symptom*" OR "Abdominal pain" OR Nausea OR Vomiting OR Diarrhea OR Diarrhoea OR Constipation OR Indigestion OR Heartburn OR Bloating OR Melena OR Distension OR Dyspepsia OR Flatulence OR "Bowel change*" OR Regurgitation OR "Acid reflux" OR Emesis OR "Irritable bowel" OR "Functional gut" OR "Functional gastrointestinal" OR "Functional bowel" OR "gut-brain interaction" OR Borborygmi OR Gurgling)
3. 1 AND 2
4. “Shift  schedule*” OR “Shift Work” OR Shiftwork OR “Night Shift” OR “Night Duty” OR “Overnight Work”     OR “Evening shift” OR “Evening work” OR  ”Late-shift” OR “Graveyard shift” OR “Third shift”  OR “Rotating Shift”  OR “Rotational shift” OR “Afternoon Shift” OR “Afternoon work” OR “Morning Shift” OR “Morning work”  OR “Night work” OR "Non day Work”  OR "Alternative shift" OR "irregular work schedule" OR “Simulated shift work” OR “Circadian Rhythm*” OR “Day Shift” OR “Irregular shift”  OR “Extended shift”  OR “Circadian rhythm* disruption*”
5. “Gastrointestinal symptom*” OR “Abdominal pain” OR Nausea OR Vomiting OR Diarrhea OR Diarrhoea OR Constipation OR Indigestion OR Heartburn OR Bloating OR Melena OR Distension OR Dyspepsia OR Flatulence OR “Bowel change*” OR Regurgitation OR “Acid reflux” OR Emesis OR “Irritable bowel” OR “Functional gut” OR “Functional gastrointestinal” OR “Functional bowel” OR “gut-brain interaction” OR Borborygmi OR Gurgling.
6. 1 AND 2

**Scopus**

1- (“Shift schedule*” OR “Shift Work”  OR Shiftwork OR “Night Shift” OR “Night Duty” OR “Overnight Work”  OR “Evening shift”  OR “Evening work”  OR “Late-shift” OR “Graveyard shift”  OR “Third shift”   OR “Rotating Shift”  OR “Rotational shift” OR “Afternoon Shift” OR “Afternoon work”  OR “Morning Shift” OR “Morning work”  OR “Night work” OR “Non-day Work” OR "Alternative shift" OR "irregular work schedule"  OR "Work Schedule Tolerance"[Mesh] OR “Simulated shift work” OR “Circadian Rhythm*” OR “Day Shift” OR “Irregular shift” OR “Extended shift” OR “Circadian rhythm* disruption*”)

2- ("Gastrointestinal symptom*" OR "Abdominal pain" OR Nausea OR Vomiting OR Diarrhea OR Diarrhoea OR Constipation OR Indigestion OR Heartburn OR Bloating OR Melena OR Distension OR Dyspepsia OR Flatulence OR "Bowel change*" OR Regurgitation OR "Acid reflux" OR Emesis OR "Irritable bowel" OR "Functional gut" OR "Functional gastrointestinal" OR "Functional bowel" OR "gut-brain interaction" OR Borborygmi OR Gurgling)

3- 1 AND 2

**Grey Literature Search**

**Clinical Trial Registry:**  <https://trialsearch.who.int>, [Home | ClinicalTrials.gov](https://clinicaltrials.gov/)

(“Shift schedule*” OR “Shift Work”  OR Shiftwork OR “Night Shift” OR “Night Duty” OR “Overnight Work”  OR “Evening shift”  OR “Evening work”  OR “Late-shift” OR “Graveyard shift”  OR “Third shift”   OR “Rotating Shift”  OR “Rotational shift” OR “Afternoon Shift” OR “Afternoon work”  OR “Morning Shift” OR “Morning work”  OR “Night work” OR “Non-day Work” OR "Alternative shift" OR "irregular work schedule"  OR "Work Schedule Tolerance" OR “Simulated shift work” OR “Circadian Rhythm*” OR “Day Shift” OR “Irregular shift” OR “Extended shift” OR “Circadian rhythm* disruption*”) AND ("Gastrointestinal symptom*" OR "Abdominal pain" OR Nausea OR Vomiting OR Diarrhea OR Diarrhoea OR Constipation OR Indigestion OR Heartburn OR Bloating OR Melena OR Distension OR Dyspepsia OR Flatulence OR "Bowel change*" OR Regurgitation OR "Acid reflux" OR Emesis OR "Irritable bowel" OR "Functional gut" OR "Functional gastrointestinal" OR "Functional bowel" OR "gut-brain interaction" OR Borborygmi)

**Preprints: Google Scholar**

1- (“Shift schedule*” OR “Shift Work”  OR Shiftwork OR “Night Shift” OR “Night Duty” OR “Overnight Work”  OR “Evening shift”  OR “Evening work”  OR “Late-shift” OR “Graveyard shift”  OR “Third shift”   OR “Rotating Shift”  OR “Rotational shift” OR “Afternoon Shift” OR “Afternoon work”  OR “Morning Shift” OR “Morning work”  OR “Night work” OR “Non-day Work” OR "Alternative shift" OR "irregular work schedule"  OR "Work Schedule Tolerance" OR “Simulated shift work” OR “Circadian Rhythm*” OR “Day Shift” OR “Irregular shift” OR “Extended shift” OR “Circadian rhythm* disruption*”)

2- ("Gastrointestinal symptom*" OR "Abdominal pain" OR Nausea OR Vomiting OR Diarrhea OR Diarrhoea OR Constipation OR Indigestion OR Heartburn OR Bloating OR Melena OR Distension OR Dyspepsia OR Flatulence OR "Bowel change*" OR Regurgitation OR "Acid reflux" OR Emesis OR "Irritable bowel" OR "Functional gut" OR "Functional gastrointestinal" OR "Functional bowel" OR "gut-brain interaction" OR Borborygmi)

3- 1 AND 2

**Preprints:**

<https://www.medrxiv.org/>, https://www.biorxiv.org/

“Shift schedule*” OR “Shift Work” OR Shiftwork AND "Gastrointestinal symptom*" OR "Abdominal pain" OR Nausea

***Table S3*** *Detailed Information of The Included Articles*

**Table S3:** Terminologies

| **Term** | **Definition** |
| --- | --- |
| Regular shift | Fixed and consistent work hours (e.g., always daytime, evening, or night). |
| Daytime shift | Working during traditional daytime “day” hours (e.g., 8 AM–4 PM, 9 AM–5 PM). |
| Rotating shift | Alternating between different shifts over time (e.g., morning → night). |
| Fortnight about | Workers change their shift schedule every two weeks. |
| Three shift system | 24-hour period split into three 8-hour shifts (morning, evening, night). |
| Dietary behaviours | Observable or self-reported patterns and practices related to food and beverage consumption, including, food quantity and quality, type of food consumed, meal timing, and meal frequency. |
| Meal regularity ^a^ | Meal pattern regularity was evaluated using a single-item measure on a 5-point Likert scale (1 = very irregular, 5 = very regular). |
| Non-standardised  self-reporting questionnaire | A self developed or unspecified tool used to assess gastrointestinal symptoms, without reference to a clearly named, validated, or previously established instrument. |
| Study-specific dietary questionnaire | These referred to the tools that used to assess diet without specific names. |
| General GI symptoms | Refers to digestive complaints or a group of GI symptoms reported collectively without specification of individual symptom types, as presented in the original analysis. |
| IBS-related symptoms | Encompasses GI symptoms commonly associated with IBS, including—but not limited to—abdominal pain, constipation, diarrhoea, and bloating. |

Abbreviations: GI, gastrointestinal symptoms; IBS, irritable bowel syndrome.

a. As assessed and defined in one of the included articles (1).

**Table S4:** Thresholds and Dose-Response Evidence for Caffeine and Alcohol Consumption and GI Symptoms

| **Exposure** | **Threshold Defined** | **Dose-Response Assessed** | **Notes** |
| --- | --- | --- | --- |
| **Caffeine** | >3 cups/day (2) | No | Increased risk of GI disorders. |
|  | >1 cup/day (3) | No | Higher GI symptom scores, marginally non-significant. |
|  | Not specified (4) | No | Associated with heartburn and bloating; no threshold given. |
|  | Not specified (5) | No | No significant association. |
|  | Not specified (6) | No | No significant association. |
| **Alcohol** | ≥3 times/week (3) | No | Higher GI symptom score, but not significant. |
|  | ≥5 glasses (women) or ≥7 glasses (men) per occasion, ≥2 times/week (1) | No | Significant association with functional dyspepsia (p=0.026). |
|  | Not specified (7) | No | Alcohol increased risk of IBS; no threshold given. |
|  | Not specified (8) | No | No association with GERD. |

**References:**

1. Jung HS, Lee B. Factors associated with the occurrence of functional dyspepsia and insomnia in shift-working nurses. Work. 2016 May 31;54(1):93–101.

2. Ahmed A and OE. GASTROINTESTINAL DISORDERS AMONG SHIFT. Egyptian Journal of Occupational Medicine . 2017;1–13.

3. Hwang SK, Lee YJ, Cho ME, Kim BK, Yoon YI. Factors Associated with Gastrointestinal Symptoms among Rotating Shift Nurses in South Korea: A Cross-Sectional Study. Int J Environ Res Public Health. 2022 Aug 1;19(16).

4. de Rijk MG, van Eekelen APJ, Kaldenberg E, Boesveldt S, te Woerd W, Holwerda T, et al. The association between eating frequency with alertness and gastrointestinal complaints in nurses during the night shift. J Sleep Res. 2021;30(5).

5. Yildiz FA, Esin MN. Self-reported gastrointestinal and cardiovascular symptoms in female Turkish nurses. Int Nurs Rev [Internet]. 2009;56(4):491–7, http://ovidsp.ovid.com/ovidweb.cgi?T=JS&PAGE=reference&D=med7&NEWS=N&AN=19930079

6. Zhou HQ, Yao M, Chen YW, Huang JY, Chen GY. Functional gastrointestinal disorders common among nurses with poor sleep quality in Shanghai, China: A pilot study. Gastroenterology Nursing. 2017;40(4):312–9.

7. Liu L, Xiao QF, Zhang YL, Yao SK. A cross-sectional study of irritable bowel syndrome in nurses in China: Prevalence and associated psychological and lifestyle factors. J Zhejiang Univ Sci B. 2014;15(6):590–7.

8. Xue J, Zhao Y, Wang Z, Ren N, Zhou C, Qin S. Rotating night shift work is associated with an increased risk of gastroesophageal reflux disease (GERD) symptoms among workers in China: A cross-sectional study. Int J Clin Pract [Internet]. 2021 Apr 15;75(4):1–6, https://search.ebscohost.com/login.aspx?direct=true&db=cin20&AN=149376414&site=ehost-live
